# Proposing an integrative, dynamic and transdiagnostic model for addictions: dysregulation phenomena of the three main modes of the predostatic mind

**DOI:** 10.3389/fpsyt.2023.1298002

**Published:** 2024-01-11

**Authors:** Bibiana Bolten Lucion Loreto, Anne Orgler Sordi, Melina Nogueira de Castro, Felipe Ornell, Eduardo Pegoraro Guarnieri, Thiago Henrique Roza, Jaqueline Bohrer Schuch, Marcos da Silveira Cima, Flavio Pechansky, Eugênio Horácio Grevet, Rodrigo Grassi-Oliveira, Lisia von Diemen, Felix Henrique Paim Kessler

**Affiliations:** ^1^Graduate Program in Psychiatry and Behavioral Sciences, Department of Psychiatry and Legal Medicine, Federal University of Rio Grande do Sul (UFRGS), Porto Alegre, Brazil; ^2^Addiction and Forensic Psychiatry Service, Hospital de Clínicas de Porto Alegre, Porto Alegre, Brazil; ^3^Center for Drug and Alcohol Research, Hospital de Clínicas de Porto Alegre, Porto Alegre, Federal University of Rio Grande do Sul (UFRGS), Porto Alegre, Brazil; ^4^Department of Psychiatry, Universidade Federal do Paraná, Curitiba, Brazil; ^5^Department of Clinical Medicine, Translational Neuropsychiatry Unit, Aarhus University, Aarhus, Denmark

**Keywords:** addiction model, transdiagnostic, risk prediction, homeostasis, self-regulation

## Abstract

Several theories have been proposed to explain the complex diagnostic aspects related to addiction disorders and their development. Recent frameworks tend to focus on dimensional perspectives of symptoms rather than categorical systems, since substance use disorders are frequently comorbid with other psychiatric and especially personality disorders. However, useful transdiagnostic models that could integrate clinical evaluation derived from neuroscientific theories are lacking. In the present manuscript, the authors propose a model based on a new paradigm, in an attempt to better explain this complex, multifaceted phenomenon. The new paradigm presupposes that emotions and behavior are a response to risk prediction. Individuals make choices and engage in actions to manage potential risks/rewards in order to seek or maintain homeostasis in their internal and external environments – a mechanism that the authors call predostatic (predictive mechanism with homeostatic purpose). The model considers three main modes of the predostatic mind: (1) Alarm Mode, activated by high and/or imminent risk prediction; (2) Seek Mode, activated by long-term risk or reward prediction; and (3) Balance Mode, a self-regulating state of mind related to low risk prediction, a soothing system and a calm state. Addiction is seen as a chronic dysregulation of organism systems leading to internalizing or externalizing phenomena mainly related to the Seek and Alarm Modes, which are persistently activated by reward and risk prediction, respectively, thus hindering Balance. Addiction neuroscience research has shown that chronic drug use or engagement in addictive behaviors can lead to neuroadaptations in the brain reward circuitry, disrupting normal balance and the regulation of reward processes. This dysregulation can contribute to persistent drug-seeking/addictive behaviors despite negative consequences. This newly proposed dynamic and integrative model, named dysregulation based on externalizing and internalizing phenomena of the three main modes of the predostatic mind (DREXI3), proposes six dysregulation dimensions with basic emotional and behavioral symptoms, such as neurophysiological alterations, impulsivity, compulsion, cognitive impairment/psychosis, mood, and anxiety/anger. In this paper, the authors explain the rationale behind DREXI3 and present some hypothetical clinical examples to better illustrate the use of the model in clinical practice. The development of this innovative model could possibly guide tailored treatment interventions in the addiction field.

## 1 Introduction

Addictive behaviors, especially drug use disorders, have become more prevalent in the 21st century. According to the World Drug Report, the worldwide number of people who reported having used drugs within the last 12 months was higher in 2020 versus in 2010 (1). In addition to the high prevalence rates, the burden of addictive disorders is significant. Around the world, deaths from substance use disorders rose from 284,000 in 2007 to 352,000 in 2017 according to the Global Burden of Disease Study (2). For instance, alcohol and drug use disorders are among the main mental health-related causes of disability-adjusted life years in the last decades (3). Even though substances of misuse have different neurochemical profiles, addictive behaviors (directed toward a drug or behavior) usually have a common phenotype.

Despite the relevance of this topic, divergences and controversies can be found in the literature regarding the nature of addictive disorders, the concept of addiction, and even the diagnosis of these conditions, which ultimately have a negative impact on the development of effective preventive and treatment interventions. In this sense, several potential strategies aimed at improving the quality of life of these patients show poor effectiveness, leading to a lack of consensus about clinical recommendations in the field. Moreover, current therapeutic interventions tend to show low compliance rates and small to moderate effect sizes even for best-practice treatment options (4–6).

Distinct theories have been proposed to explain the mechanisms that lead to addiction. Neurobiological and neuroimaging research has drawn attention to biological theories, with evidence supporting the role of rewarding neural pathways, especially the ones related to dopamine, in seeking behavior and reward (7). Conversely, reflexive choice theories state that individuals can consciously choose to engage in addictive behaviors (8, 9), whereas the self-medication hypothesis, proposed by Khantzian, claims that individuals are predisposed to addiction because of negative affect states and comorbid psychiatric disorders; in this mental scenario, the effect of the drug of choice - as well as the individual’s perception of some type of control over his/her inner feelings would balance the prevailing negative emotion (10). This latter hypothesis has been reviewed by its author, associating specific substances with psychiatric comorbidities, with conflicting evidence (some supporting and some weighing against the theory) (11).

Some authors have tried to explain addictive behaviors and emotions from different perspectives. The automatic processing theories, for instance, propose that addictive behaviors can be acquired without self-conscious intentions (12–15). Goal-focused theories, in turn, have tried to explain addiction by relating its development with the seeking of positive rewards or the avoidance of negative emotions – these include the drug withdrawal theory, which defines addiction as the physiological adaptation and resulting withdrawal symptoms that occur as a consequence of drug use (16). In recent years, theories focusing on the individual have tended to describe addiction as the result of a combination of factors, mainly environment and internal characteristics – therefore, they are called integrative theories (17–19). One example is the PRIME theory, by Robert West, which attempted to integrate features of different models of addiction in a single model. According to that model, motivation should be a common aspect in addiction theories (20).

Another category of theories tries to explain addiction at a population level. Social network theories, for example, propose that the prevalence of addiction in a population is related to the connections between individuals with and without addictive behaviors (21, 22). Economic models state that addictive behaviors can be predicted by functions (23, 24). Communication theories focus on marketing activities as having a great influence on patients with addiction, which are considered “consumers” (25, 26). Furthermore, the organizational system model understands addictive behaviors as an interaction between different elements of a society (27, 28). In addition to a dispute across research lines suggesting that the phenomena of addiction are related to brain disease, choice disorder, or a learning process (29), the diagnostic criteria of addiction in existing manuals have also raised discussion.

Currently, the clinical diagnosis of substance use disorder is generally based on categorical entities originating from classification systems (30). Even though classification systems such as the DSM-5 and ICD-11 have made significant contributions in terms of providing reliability for diagnostic procedures - which ultimately serve as the basis for clinical assessment, treatment-decision, scientific investigation, and public health policies, they are also often the object of criticism. Much has been discussed about the potential of medicalization of aspects and issues of daily life. Moreover, categorical diagnostic entities in those classification systems are usually not based on strong scientific evidence or neuroscientific concepts (30, 31). Recent studies have examined the limitations of categorical systems in assessing psychopathology (32), which also impacts the field of addiction. Translating diagnostic criteria of addiction into terms of quantitative behavioral processes could expand the possibilities of how this disorder is understood and treated (33). The Research Domain Criteria (RDoC) framework was especially designed for pathophysiology research (34), while the Hierarchical Taxonomy of Psychopathology (HiTOP) system seeks to improve the organization of psychopathology, phenomenology, illness course, and treatment response, both in research and clinical practice (35). Both these dimensional approaches have strengths that could be complementary and promote the development of new frameworks (36).

When examining the relationship between different types of addictive disorders, i.e., behavioral and substance addictions, more similarities than differences can be found. Both types of addiction tend to emerge in late teenage years or early adulthood and follow a course of lapses and recoveries. The risk factors are also similar, potentially including adverse childhood experiences, neurobiological dysregulation, low distress tolerance, and emotional dysregulation. Moreover, both behavioral and substance addictions share common clinical processes that may be targeted in treatment. Nevertheless, because categorical classifications have proven insufficient, new frameworks tend to adopt a symptomatologic perspective (37).

Bickel et al. emphasize that distinct theories may each provide a perspective on how to conceptualize addiction, but the disorder may be the result of different processes, or one symptom may be caused by distinct mechanisms, which poses a diagnostic approach challenge (33). Some other authors have been pointing to a translational crisis in the addiction field: although basic research has identified some neurological basis for addiction, the impact of these findings on clinical practice has been limited (38, 39). Research with animal models self-administering drugs has been conducted for many years, aiming to improve the understanding of a biological basis of addiction (40). However, the advances in basic research have not led to equivalent developments in therapeutic strategies. This translational failure shows the need for changes in this research field as well as in other areas within psychiatry (41).

It becomes fundamental to have a well grounded conceptual model that contemplates these patterns of emotions, thoughts, and especially behaviors. In the present paper, the authors propose a novel theoretical-clinical model to explain the phenomena of addiction from a more integrative perspective. The proposed model will be presented and its fundamental aspects discussed in light of the most relevant theories and recent paradigms in science.

## 2 The predostatic mind

The main purpose of living beings is to achieve homeostasis, which is defined as a self-regulating process by which physiological systems maintain stability while adapting to changing external conditions (42). Cannon suggested that homeostasis is the summit of countless years of evolution, by which the body reacts to changes in the environment with emotional and behavioral responses (43). In other words, living systems evolved to maintain a stable internal environment in order not to be destroyed by the forces surrounding them. It is clear that homeostasis is not static; rather, it is a dynamic self-adjusting process mediated by stimuli and internal and/or behavioral responses (44).

Homeostasis is accomplished by mechanisms of feedback and feedforward and could explain self-regulation in biological systems (42, 45). Feedback occurs when outputs of a system are routed back as inputs in a closed loop structure. The past actions of a system are fed into the system to control future actions, acting as the system’s memories. Negative feedback, in the context of physiological homeostasis of biological systems, is a mechanism that counteracts deviations from a desired state, in order to maintain stability. Positive feedback is characterized as a positive loop gain that produces growth processes. While negative feedback tends to reduce fluctuations in the output, minimizing the effects of disturbances, positive feedback tends to lead to instability via further disorganized behavior. The other mechanism utilized during the homeostasis process, namely feedforward regulation, occurs when the system responds to environmental disturbances in a pre-defined way, adjusting itself before any changes have actually occurred. It requires previous information or memory about the content and magnitude of the stimulus. In living organisms, these memories are provided by learning and experience (42). Survival also depends on the conservation of our body’s integrity, and for that it is essential to actively maintain our internal environment – homeostatic equilibrium.

The search for balance is complex in animals – especially humans – and difficult to explain. In this sense, terminology adjustments are needed to facilitate the use of a model in clinical settings or even to describe maladaptive chronic responses that could be considered mental disorders. It is relevant to understand and discuss the mechanisms that living beings use to achieve balance, and we will use the concept of mind as the resulting phenomena of all systems in an organism doing predictive work together to reach this goal. Recently, a new perspective has emerged in the debate involving mind and cognition, according to which the brain and the whole nervous system essentially work as a probabilistic prediction engine, dedicated to the task of minimizing the disparity between how it expects (predicts) the world to be and the evidence presented by the sensory stream (46). The predictive mind uses its knowledge of patterns and perceptual experiences to make predictions about events (47). Therefore, in this paper, we use the novel expression “Predostatic Mind” to better describe the predictive mechanisms by which sensoperception and animal systems evaluate environmental stimuli and try to achieve balance.

According to current theories, besides emotional states and thoughts, behavior is also a homeostatic response to environmental stimuli, and goal-directed behaviors, such as eating or sleeping, may result from predictions. We can think about this in a bidimensional way, where positive and negative predictions may occur, resulting in seek or threat response behaviors. Responses such as fight, flight, and freeze can be seen as a temporary disruption of normal processes aiming at balance. The body’s focus shifts from maintaining its internal balance to responding to the immediate threat or stressor. Once the threat is resolved, the body aims to return to homeostasis by restoring normal physiological functioning. This would be the case in less complex organisms that rely on a simpler sensory apparatus and therefore display a limited number of behaviors. In humans, however, the brain is the main hub of information processing, and the nervous system plays a crucial role in coordinating and integrating actions performed by different parts of the body. Here we must add that all systems operate in the context of the organism as a whole (48). Using environmental cues, stored information (memory and learning) and situational demands, our brain starts calculating the risk associated with the context in which the organism is found. It scans our interior and exterior environments all the time, generating interoceptive and exteroceptive maps (49). According to the predicted risk – low or high, short- or long-term, avoidable or not –, the mind will operate in different modes that are related to patterns of responses in order to achieve goals, such as fight and flight behaviors, to guarantee survival. Different modes will then trigger different responses that are appropriate for each scenario. With regard to homeostatic responses, emerging models have tried to simplify the description of motivations behind animal behaviors. Gilbert, for instance, proposes three main systems of emotional regulation: drive, threat, and soothing. While the latter is commonly related to homeostasis, the systems involved in drive and threat response behaviors are also ways of achieving it (50).

**Figure 1:**
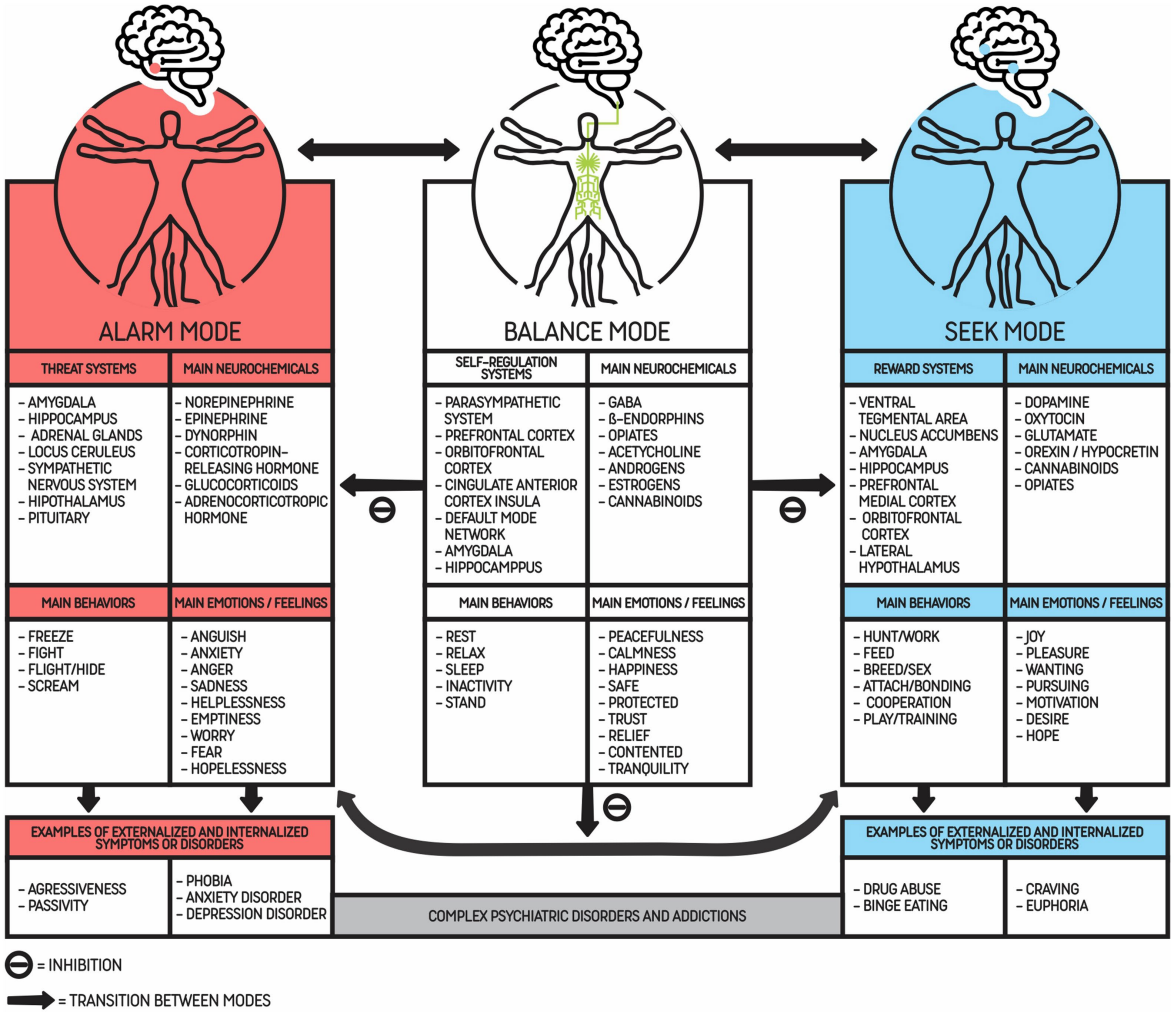
Three main modes of the predostatic mind and its main systems, behaviors, emotions and neurochemicals. This figure represents the three modes of the mind: Alarm Mode, Balance Mode, and Seek Mode. Each mode is characterized by the most known associated systems, neurochemicals, behaviors, and emotions, although some of these phenomena can be activated (inhibition or stimulation) in other modes as well. Behaviors are considered externalizing phenomena, while emotions and thoughts are associated with internalizing phenomena. Each mode is activated according to risk prediction in a dynamic way. The main usual target is to keep the organism in Balance Mode. It is relevant to highlight that culture or scarcity could push people to increase activation of the Seek Mode. The arrows indicate possible transitions between modes through stimulation and/or inhibition of systems. Worthy of note is the fact that in complex animals, such as mammals, especially *Homo sapiens*, it is common to report emotions and behaviors from different modes at the same time. When internalizing and externalizing phenomena become maladaptive or excessive from a social/cultural perspective, they can be considered symptoms that could lead to a psychiatric diagnosis of mental disorders, in particular addictions.

The present paper proposes a model in which addiction is seen as a chronic and maladaptive response of the predictive mind while trying to achieve balance, leading the individual to compulsively seek the same rewarding stimuli (e.g., drug, video game, pornography), despite acute and long-term impairments. Because we want patients to understand the proposed model, the Modes of the Mind will be reduced to three and will be referred to as Alarm Mode, Seek Mode, and Balance Mode. Each of these modes will activate specific neurobiological systems and neurochemicals, as represented in Figure 1, in varied ways. However, the description and explanation of how neurobiological systems activate the modes are not within the scope of this manuscript due to lack of space.

### 2.1 Alarm mode (threat response systems)

According to the present model, the mind will operate in Alarm Mode when the (imminent and/or severe) risk prediction increases and leads to an increment of stress levels. The disruption of mental prediction involves structures such as the amygdala, the hypothalamus, and the habenula. As the name implies, this mode will be activated when the organism faces a threat, and it will trigger stress-coping behaviors that include freezing, screaming/asking for help, fighting, or flighting. When threat is detected, the sympathetic and parasympathetic branches of the autonomic nervous system are activated simultaneously, enabling the fast onset of defensive freezing and fight-or-flight responses.

**Figure 2:**
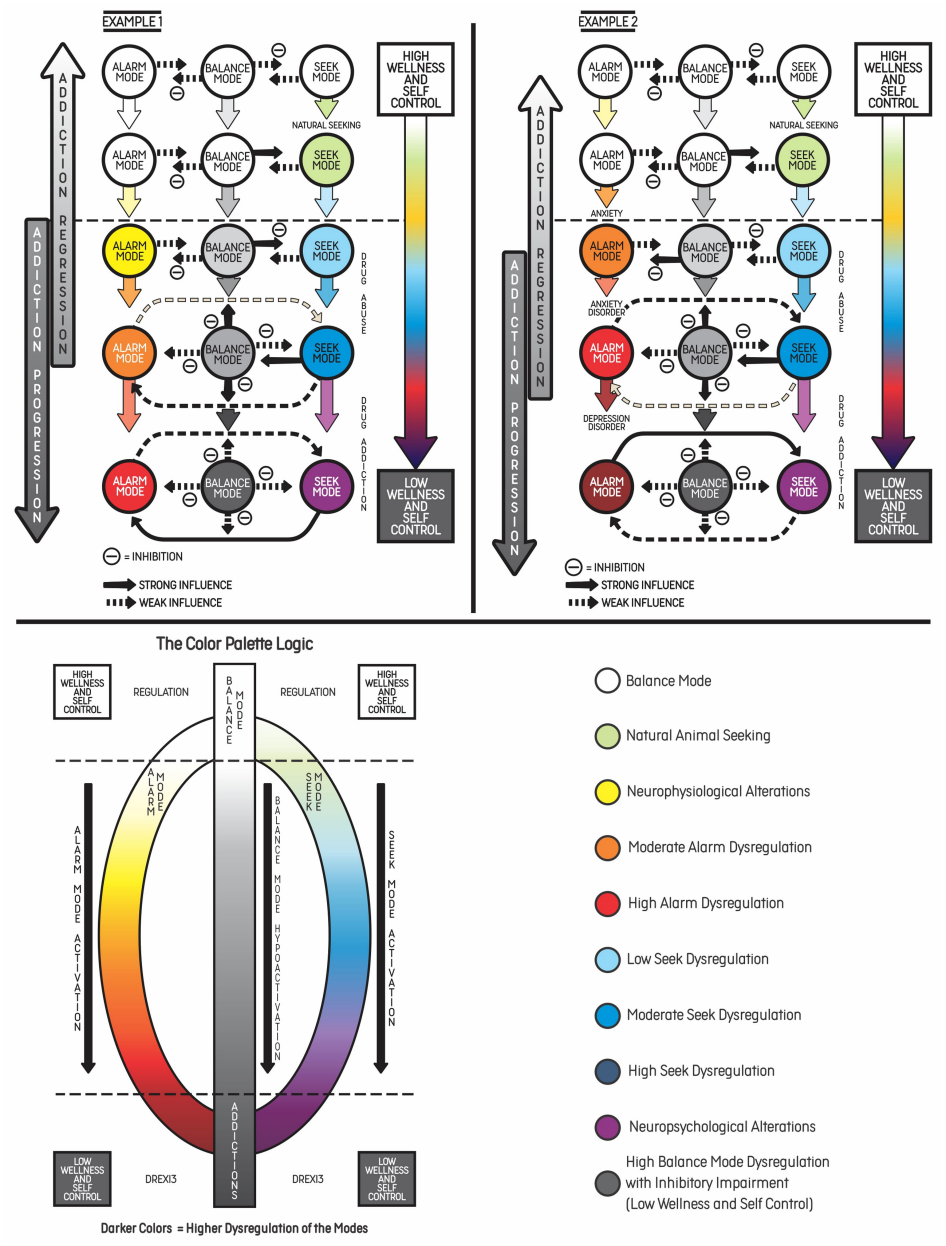
Two examples of addiction progression based on increasing dysregulation of psychobehavioral phenomena related to the three main modes of the predostatic mind. Example 1: This example illustrates the progression of addiction in young patients without psychiatric comorbidities who have been experimenting with cocaine at an increasing consumption rate over time, activating the Seek Mode and replacing natural seeking behaviors, such as sleeping, eating or having sex, with drug use. Chronic drug use can lead to withdrawal symptoms, which are related to the Alarm Mode, resulting in negative emotions or even psychiatric symptoms. According to the DREXI3 model, these patients were initially in Balance Mode, with high levels of wellness and self-control, but this chronic cycle with hyperactivation of the Seek and Alarm Modes led to addiction and long-term consequences. When a reinforcing stimulus is present (e.g., video game or the use of psychoactive substance), dysregulation of physiological functions may occur (appetite, sleep, energy, libido) as well as hyperactivation of the Seek Mode, with consequent memory and future salience of this stimulus (craving). This may lead to increased impulsivity and facilitate progression to substance abuse in individuals with a genetic predisposition. Consequently, the Alarm Mode and the Seek Mode become progressively more dysregulated, as represented by the evolution of colors. The high dysregulation of the modes tends to sustain the addictive process, creating a coping habit in order to face inner or outer stressors. On the right side of the figure, the color palette represents the logic of hyperactivation of the Alarm and Seek Modes, or hypoactivation of the Balance Mode (from white to gray). Example 2: This example shows vulnerable patients who present chronic anxiety or depressive symptoms and start to increase alcohol consumption in order to cope with negative emotions and stress (Alarm Mode hyperactivation). In this case, the Seek Mode is activated and leads to substance use in order to achieve balance. However, chronic stress and alcohol consumption predisposed the patients to develop addictive behaviors, which progressed to mental disorder as a result of emotional dysregulation and inhibitory control impairment.

When in Alarm Mode, the individual is vulnerable to imminent or severe risk prediction. It occurs, for example, when a predator is threatening an animal or its offspring. The threatened animal enters the Alarm Mode, which demands a behavior of fighting its opponent. Flight and freeze are other possible responses to achieve self-regulation (51) (or Balance Mode in the proposed model). In the context of drug addiction, the Seek Mode may be activated by stressors that are not solved by alarm responses and lead to drug use in order to achieve balance. However, once the effect of a given substance ends, withdrawal symptoms may activate the threat response systems (or Alarm Mode) again, maintaining addiction. This example is illustrated in Figure 2 and will be discussed in more detail below.

### 2.2 Seek mode (reward systems)

The Seek Mode is usually defined as the search for pleasant and hedonic stimuli (e.g., psychoactive drugs, sexual and social experiences) that can lead to positive emotions and well-being. However, there is robust evidence that behaviors associated with this mode, such as reproduction and cooperation, are also homeostatic or advantageous for individuals or groups in response to environmental predicted risks. From a neurobiological point of view, this mode is associated with the mesolimbic dopaminergic pathway. It is related to the seeking system described by Wright and Panksepp, namely, a primitive neural system focused on energetic search or conservation of resources among mammals. For example, when an animal is starving, the reward system (Seek Mode) is expected to lead to a highly “obsessive” and focused search for food. Searching for safety and sex is also part of this scenario (52). The Seek Mode includes positive sensations of reward when its demands are fulfilled and the animal can self-regulate again.

This mode can also be associated with success in social and professional (equivalent of hunting) aspects of life. From an evolutionary perspective, the great role of the positive emotions in the Seek Mode would be to improve physical, intellectual, and social abilities so as to promote better adaptation in the long term. In this sense, behaviors related to social connections in some mammals may be linked to positive affect, which is hypothetically associated with hormones such as oxytocin. The orbitofrontal cortex encodes subjective affective valence, which is related to reward in response to a variety of stimuli, whereas the anterior cingulate cortex and its connections with limbic structures, like the amygdala and the hippocampus, are involved in this homeostatic process through top-down control and emotional regulation. The insula is also involved in this context, as it integrates interoception and emotional conscience (53).

### 2.3 Balance mode (soothing systems)

The Balance Mode is associated with low risk prediction in present and future perspectives, i.e., it is in place when the individual experiences a state of self-regulation. This is a positive affect state, related to sensations of calmness, rest, contentment – and therefore, behaviors such as sleeping, resting, and relaxing. Depue and Morrone-Strupinsky have demonstrated the existence of a peculiar positive affect system linked to calming, resting, and contentment – a state of quiescence where one is neither under threat nor in a seeking or achieving state of mind, i.e., both the drive and the threat response systems are characterized by calmness (54). Once a goal has been achieved and the animal is not under threat, drive systems need to be “turned off” to rest, produce quiescence, and balance energy expenditure. This system is linked to endorphins and the activation of the parasympathetic nervous system, which is sometimes called a “rest and digest” system (55).

Human and animal research has focused on positive states, which sometimes can be defined as a sensation of calmness and well-being associated with the Balance Mode. The structures involved in well-being include the left prefrontal cortex, which seems to process positive affect and emotions. Opioid release in the amygdala has been reported to diminish its activity during positive emotional experiences in humans (56). Furthermore, the possible role of the Default Mode Network (DMN) in internal regulation should not be overlooked: its involvement in goal-directed tasks like working memory and “introspective behaviors” may be interpreted as an improvement to the internal prediction model. In general attentional states, the DMN would hypomodulate and promote activity in other brain regions responsible for focusing on the external environment. In our homeostatic model, the DMN could hypothetically be related to an internal regulation state, in which the mind works to improve prediction by revising information and memories to adjust predictive models and increase the chance of building more adaptive behavioral responses in the future (57).

## 3 Addiction as dysregulation based on externalizing and internalizing phenomena of the three main modes of the predostatic mind (DREXI3)

The addiction model proposed by the authors, named dysregulation of the three main modes of the predostatic mind (DREXI3), involves externalizing (behaviors) and internalizing (thoughts and emotions) phenomena. The model describes addiction as a consequence of chronic dysregulation of regulatory systems associated with the three modes described above. Figure 2 shows two types of addiction progression toward a mental disorder according to the idea of different degrees of activation of the modes of the mind. In both examples, the patients begin with greater balance and self-control and progress to significant activation of the Alarm Mode (emotional suffering and threat response behaviors) and the Seek Mode (seek behaviors, although with less positive emotions related to the drug), resulting in low balance and high impairments. According to our model, an interaction between (or simultaneous activation of) the Seek Mode and the Alarm Mode may occur when seeking becomes chronic and leads to a compulsive behavior that fails to achieve balance. The individual may not obtain the desired outcome and experience a state of withdrawal or negative affect (irritability, dysphoria, alexithymia, stress, and lack of motivation for natural rewards).

**Figure 3:**
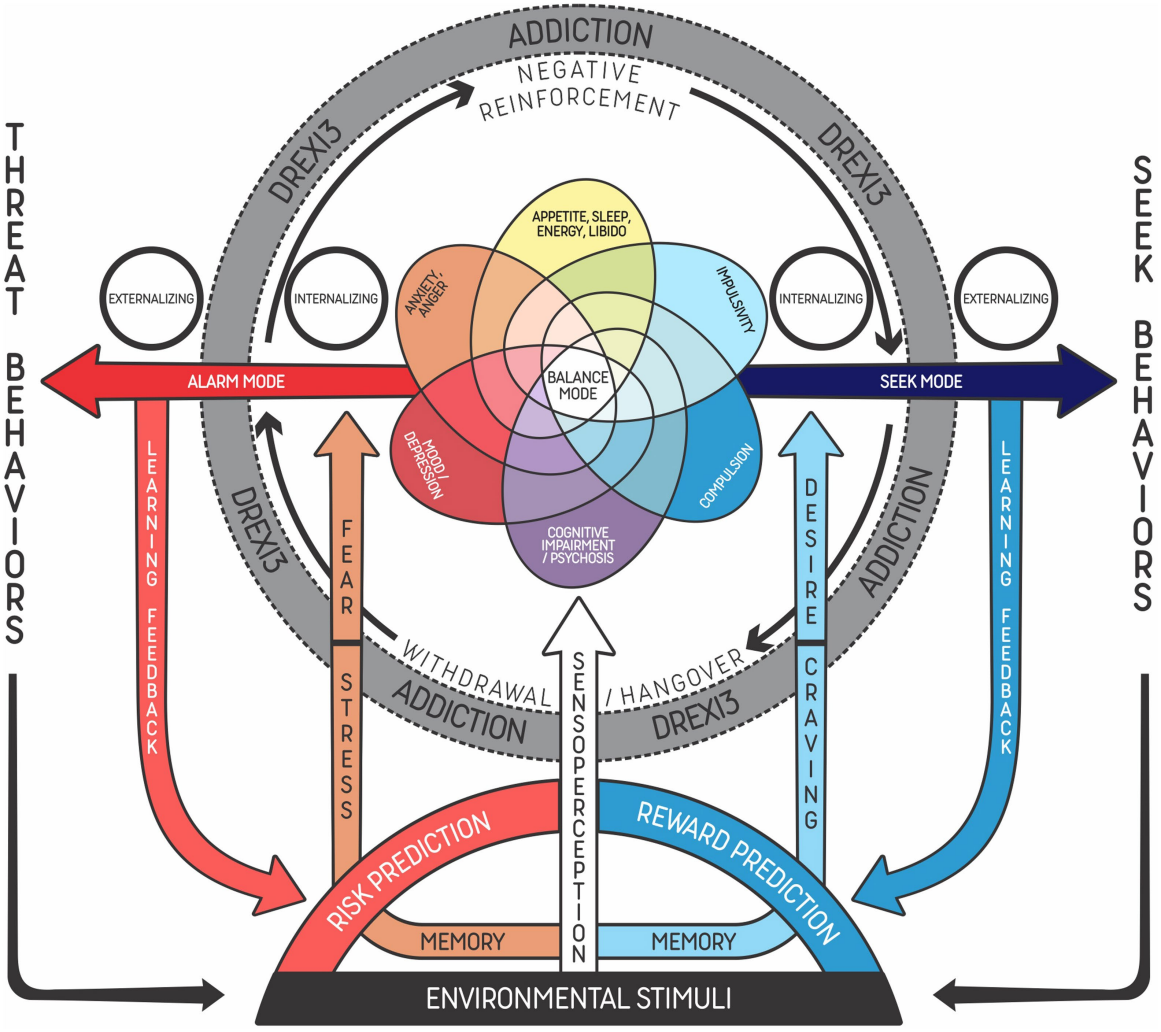
Addiction as dysregulation based on externalizing and internalizing phenomena of the three main modes of the predostatic mind (DREXI3). Representation of the functioning of individuals with an addictive disorder undergoing a severe situation, in which the mind tends to fluctuate between the Alarm Mode (in red) and the Seek Mode (in blue), struggling to keep balance. As the condition evolves, memories of risk associated with withdrawal symptoms are reinforced (negative feedback), and this prediction then triggers stress responses, leading to seeking behaviors that aim to achieve self-regulation. Due to strong memories of reward (learning feedback) related to the drug, the predictive mechanism activates substance craving as a means of reducing risks. This reinforces the emotional and behavioral cycle (in gray) associated with the increased activation of systems related to the Alarm and Seek Modes (allostasis) and decreased use of systems related to the typical animal Balance Mode (homeostasis). Fearful learning feedback is triggered in the Alarm Mode, leading to a prediction of risk and reactions of fight, fight, or freeze (stress). Conversely, rewarding learning feedback is increased every time regulation is easily achieved with strong stimuli (e.g., drug, video game, social media). During this process, there is an increase of psychopathological phenomena or symptoms in many domains of the transdiagnostic model, according to the type, frequency and severity of substance use.

In the examples, addiction progression results from processes involving chronic and systematic (frequent) activation of the Alarm and Seek Modes, especially in individuals who present some risk factors, such as impulsivity or stressful life events. In some situations, the Alarm Mode may lead to activation of the Seek Mode to achieve internal regulation, particularly when the problem is difficult to solve or when risk prediction is not imminent or related to imminent survival. Drug use only produces a short-duration balance and rarely helps to achieve an adequate response to environmental stimuli, which in turn may trigger the Alarm Mode again. There is a neurobiological basis showing how drug use can affect the prefrontal cortex and impact executive function and decision-making, maintaining addictive behaviors and further impairing self-control in the long term (58). Figure 3 shows the fluctuation between the Alarm and Seek Modes.

Imaging studies conducted with addicted humans have reported that reward brain circuits become less sensitive to natural rewards during states of negative affect. Both the reduction of reward and the increase of stress are cues to the Alarm Mode. In the context of substance use disorders, incentive salience can be developed, linking a cue, e.g., a stressor, to drug use. This state could be interpreted as craving, which consists of worry and anticipation that induce the Seek Mode to find rewarding stimuli in order to achieve regulation. This cue-induced craving is mediated by activation of the prefrontal cortex, including the dorsolateral prefrontal cortex, the anterior cingulate gyrus, and the medial orbitofrontal cortex (58). While in the threat system the outcomes include fight and freeze, aggravation or hyperactivation of the Seek Mode can lead to intoxication and even death by overdose in cases of substance use disorders.

### 3.1 Risk prediction, negative reinforcement and addiction

Previous theories of addiction emphasize the search for regulation when facing negative emotions as a determinant factor in addictive behaviors. Jacobs defines addiction as an acquired state of dependence aimed to alleviate stress. His general theory of addiction proposes two determinants for the development of these disorders: existence of an excessively depressed or agitated chronic physiological state; and reaction to adverse experiences early in life. According to that author, the process of addiction would develop in three phases: stage I would be the discovery of the addictive behavior as a potential relief of negative emotions; in stage II, the positive reinforcer of addictive behaviors would make them become compulsive and resistant to change; finally, in stage III, the individual would keep the addictive behavior to avoid negative emotions, in spite of the harm associated (37, 59). The DREXI3 model follows a similar logic, reinforcing the search for balance and self-regulation as a determinant for maintaining the addictive behavior: the Alarm Mode represents an answer to environmental stimuli that cause stress and risk prediction; the addictive behavior then arises with the aim of relief, leading the individual to enter Seek Mode; due to reward prediction and avoidance of negative emotions, the behavior becomes progressively compulsive.

Other theories recently published propose a transdiagnostic treatment model for both substance use disorders and behavioral addictions, considering the similarities between these pathologies. For instance, the addiction components model explains addictive disorders based on these common features, describing six main components: salience, which can manifest as craving; mood changes associated with relief of negative affects; tolerance; abstinence symptoms; conflicts due to the addictive behavior; and relapse (37). Theories like this were also taken into consideration when developing DREXI3, by emphasizing the role of craving (strong and recurrent reward prediction) and the search for self-regulation when facing negative emotions or other internal dysregulations.

Addiction has also been analyzed from an evolutionary perspective. Some characteristics of the addictive process tend to be considered exclusive of mammals and even of humans, due to their superior cognitive abilities. The neurosystems involved in the search for reward were shaped across evolution to reinforce some behaviors (60). However, evidence of other animal species that also consume psychoactive substances suggests that the action of the involved systems is not restricted to humans (61, 62). For the scope of DREXI3, addiction is seen as a result of phenomena related to neural systems that are evolutionarily relevant for survival and balance.

The search for a homeostatic hypothesis corroborates other addiction theories that also focus on the search for regulation (10, 16, 20). Both clinical practice and scientific evidence suggest that negative affect states predispose to substance use as a way to alleviate unpleasant emotions and bring the individual to a point of balance (14, 58, 63, 64). Actually, this point of balance is maintained for a short period of time, automatically returning to the negative valence state observed previous to drug use. According to Koob, frequent use of psychoactive substances induces several neurochemical changes derived from the tolerance associated with sustained drug use, leading to what he calls hyperkatifeia, a chronic anhedonic state related to an increased threshold for pleasant emotions (63). Other authors suggest that the negative emotional states leading to substance use are due to psychiatric comorbidities, like mood and anxiety disorders. Substance use, in this sense, would be a way of self-medication (10). Social psychiatry and psychology researchers correlate the impact of environmental factors (poverty, hunger, war) with the activation of a chronic stress state that could lead to substance use and the associated consequences (65–68). Finally, many authors point out specific elements as being the cause of addiction, although the behavior pattern can originate from different factors (69–71). In conclusion, any negative valence state can be understood as risk prediction, and drug use would be an attempt to return to balance. Repetition of the same behavior reinforces the memory of reward and the prediction of a homeostatic result, consolidating the cycle. Individual genetic and environmental features, as well as the action of each substance in the central nervous system, can influence the process. For this reason, the development of addiction can occur at different time periods for each individual.

Internalizing disorders, such as mood and anxiety disorders, are directly related to feelings of hopelessness, fear, and a state of stress. Individuals with these comorbidities are four times more likely to become addicts and are more prone to relapse (72). There is also an important association between externalizing symptoms and personality disorders, as impulsivity limits the individual’s ability to control their own behavior, and the predominance of emotional dysregulation states leads the individual to seek addictive behavior as a relief of negative states (73).

Conversely, when risk prediction concerning possible consequences of addictive behavior increases significantly, there is a trend toward protective behavior instead of substance use. This process, mediated by prefrontal structures, may suppress the addictive cycle and promote a change towards recovery. However, risk perception, too, has different components and may be influenced by type of addictive behavior (74, 75).

## 4 Six main transdiagnostic dimensions of dysregulation derived from DREXI3 model

**Figure 4:**
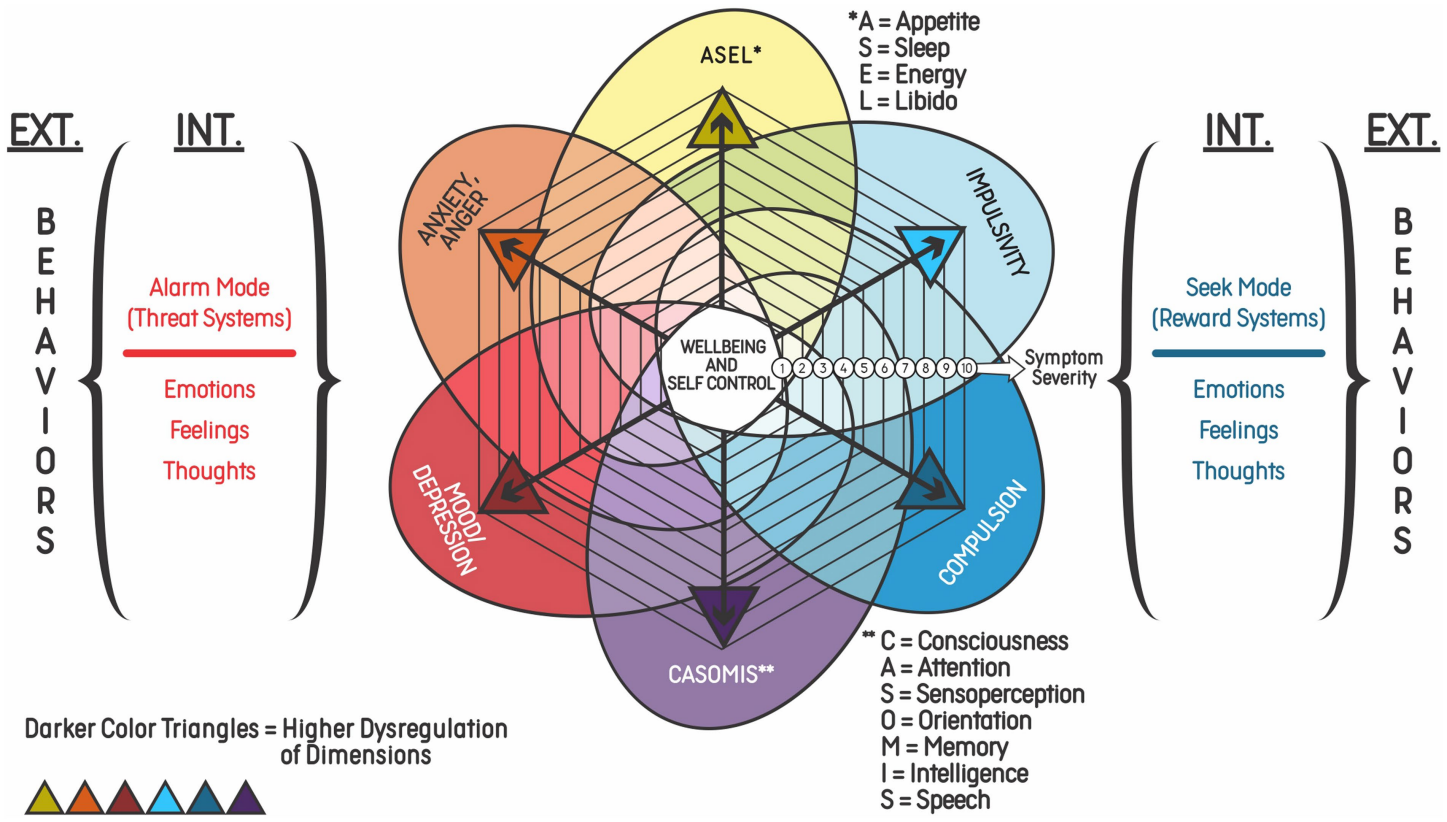
Six transdiagnostic dimensions of the DREXI3 model of dysregulation leading to externalizing and internalizing symptoms. Representation of the six dimensions of symptoms possibly observed in addiction: neurophysiological alterations, impulsivity; compulsion; cognitive impairment and psychosis; mood disorders/depression; and anxiety/anger. Each dimension is represented by a color and the severity of symptoms increases with the distance of a point from the center, which is a state of wellbeing and self-control. Activation of the Alarm and Seek Modes can cause both externalizing and internalizing symptoms, as represented by “EXT.” and “INT.” The severity of symptoms is related to the intensity of dysregulation. While emotions/feelings associated with the Alarm Mode are usually considered to be of negative valence and risk prediction, emotions/feelings in the Seek Mode are usually considered to be of positive valence and reward prediction.

In order to facilitate the identification of mind predictions and responses (emotions, thoughts, and behaviors) related to the DREXI3 model, the authors designed a reductionist transdiagnostic radar chart illustration containing the six main dimensions of externalizing and internalizing phenomena usually found in animals with addictive behaviors (Figure 4). The model highlights the differences between internalizing and externalizing phenomena associated with each of the three modes, and their evaluation could be helpful to detect changes across these modes. The terms “internalizing” and “externalizing” were originally used to describe groups of symptoms in children and adolescents and became well accepted classifications for behavioral disorders. These concepts also have an empirical basis and are useful for both clinical practice and research. While the internalizing group comprises depressive, anxiety and somatic disorders, the externalizing group represents conditions characterized by impulsive and compulsive behaviors, including addiction (76, 77). In the DREXI3 model, addiction usually presents both internalizing and externalizing symptoms, with two hyperactivated modes (Seek and Alarm Modes) and one hypoactivated mode (Balance Mode).

Figure 4 presents the six dimensions of symptoms possibly observed in addictive disorders, which are the same found in Figure 3, namely: alterations in appetite, sleep, energy, and libido; anxiety/anger; impulsivity, mood/depression; compulsion; and cognitive impairment/psychosis. This proposal is based on the latest classifications and theories on the subject (e.g., DSM-5-TR). Among the diagnostic criteria listed, some are directly related to the patient’s inability to control substance use. For instance, use of excessive amounts of drug, difficulty to cease use, and time spent with use would be manifestations of the compulsion dimension. Use of a substance in situations that can offer risks or when substance use has physical or psychological complications would be included in the impulsivity dimension. Craving, induced by reward memories, would be activated by predictive mechanisms that signal the need to use the drug again as a way of reducing risks/achieving balance. Finally, abstinence symptoms would be associated with risk prediction that triggers stress responses, leading the individual to self-regulating seeking behaviors (77).

**Figure 5:**
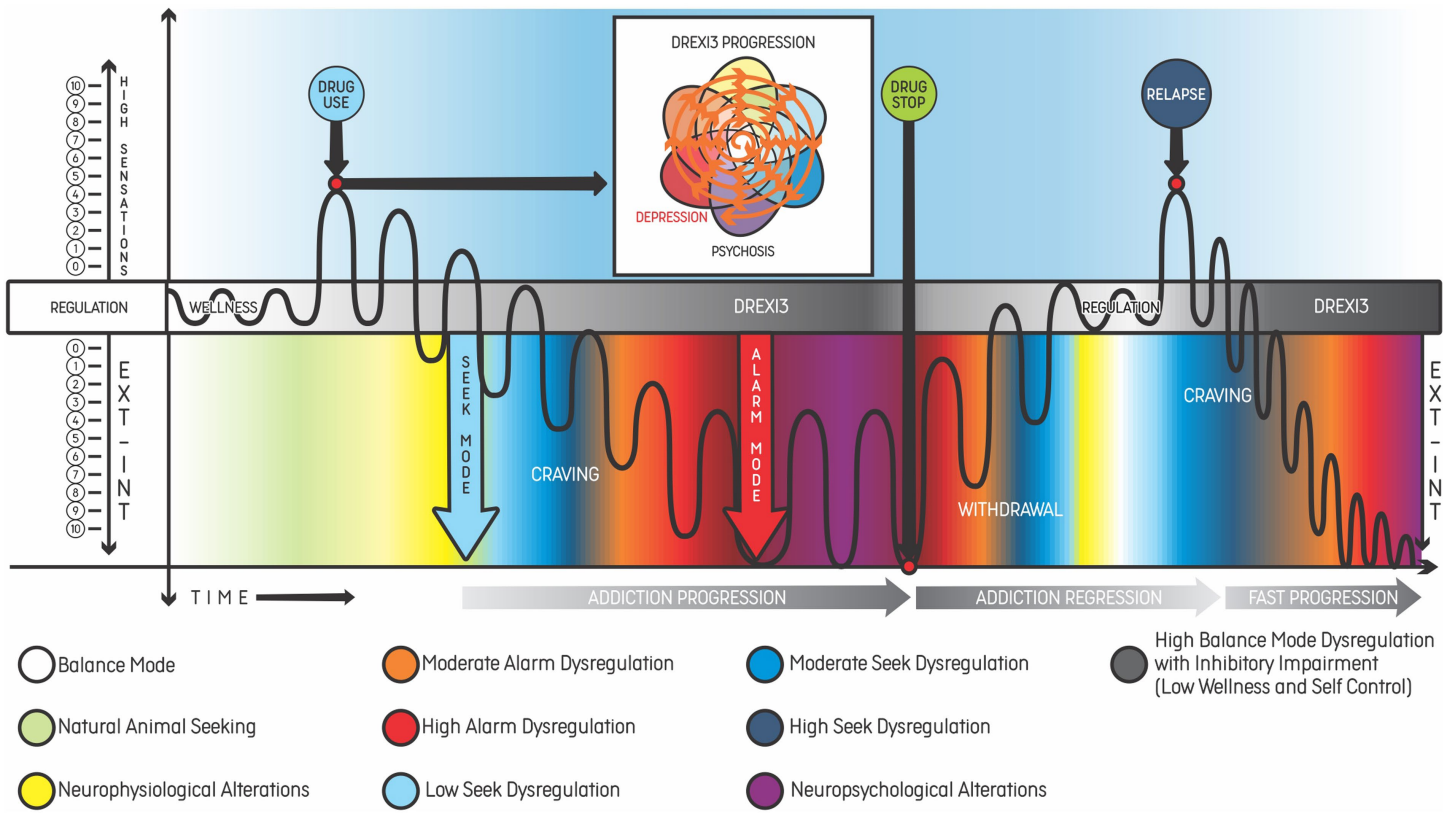
Example of addiction progression according to the DREXI3 model, beginning with drug experimentation in an individual with genetic predisposition. The progression of a healthy individual (patient 1 from Figure 2) after experimenting with cocaine and the relationship between disorder progression and the worsening of symptoms represented by the same colors of the DREXI3 model dimensions. The line represents adaptive (white/gray bar in the middle) and maladaptive responses (colored phenomena according to DREXI3). In the white box above the bar, centrifugal spiral arrows show the increase of psychiatric symptoms in all dimensions as the addiction disorder progresses, with decreased well-being and self-control until drug use stops. After the withdrawal phase, most of the psychiatric symptoms tend to improve towards regulation or homeostasis, provided no other relevant psychiatric comorbidities are present. Continuous drug use leads the patient to waves of an unstable regulation of the homeostasis, with emotional and behavioral dysregulation (suffering, inadequate and poor responses), which is also considered an allostatic state. Black “waves” in the figure represent drug consumption and its effects - such as internalizing (INT) and externalizing (EXT) phemonena. The variation of the waves represents the intensity of use - as well as its corresponding effects. The greater the dysregulation and the distance from the homeostatic state, the greater the allostatic load related to the disorder. For instance, in an individual diagnosed with alcohol use disorder, the mechanisms of addiction are installed, meaning that, after relapse, the process of dysregulation phenomena will occur more rapidly, as we can see in the final lines of the figure.

**Figure 6:**
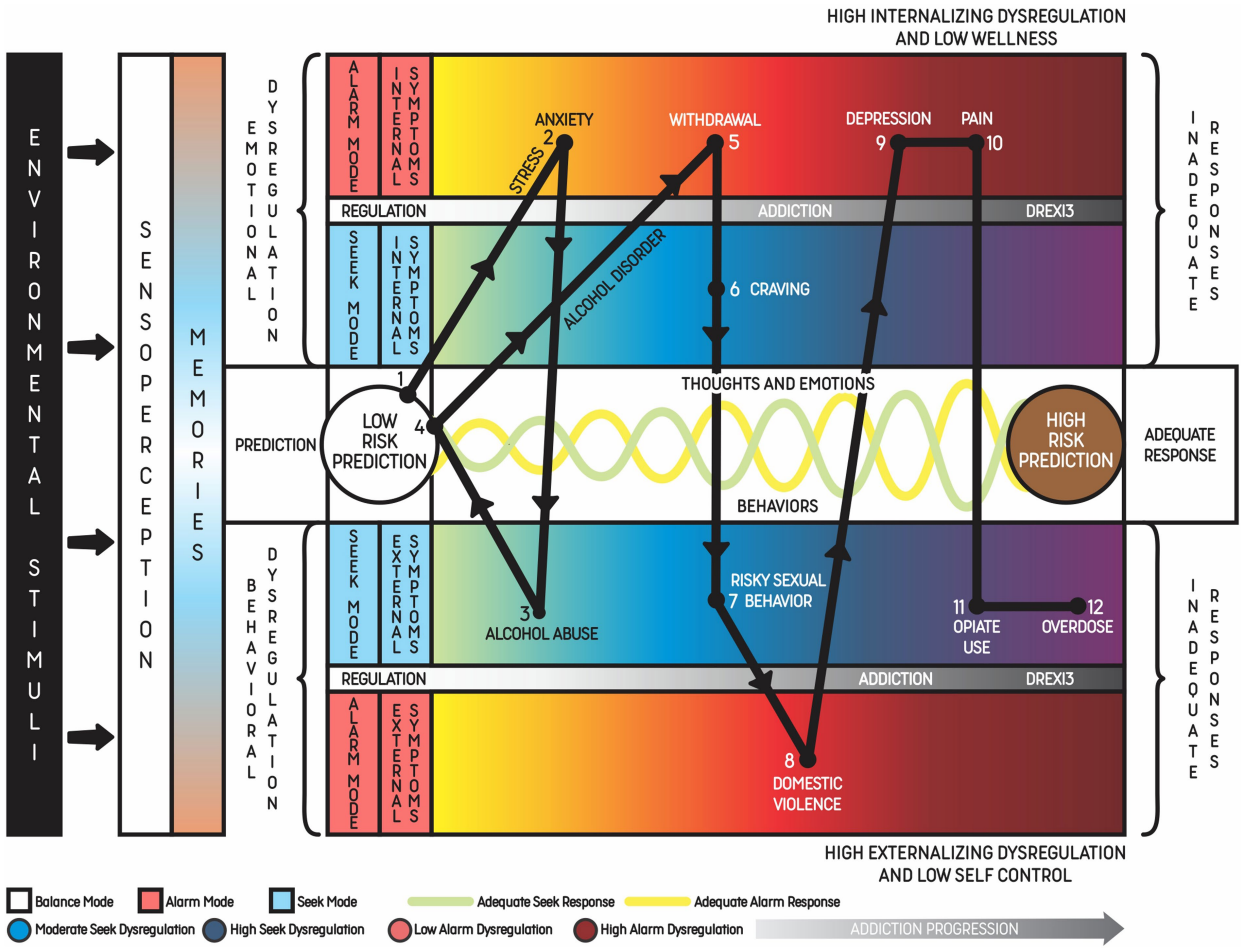
Example of alcohol use disorder progression over 10 years with increasing externalizing and internalizing phenomena until death by overdose. A 10-year natural progression of alcohol use disorder that evolved with internalized and externalized symptoms of the Alarm (red scale) and Seek (blue scale) Modes, beginning with comorbid anxiety disorder. This example represents young patients who are healthy (state of low-risk prediction) but start to face chronic stress due to financial problems, which activates the Alarm Mode and increases activation of the Seek Mode. Constant activation of the Alarm Mode leads to anxiety disorder. The patient keeps using alcohol to achieve the state of low-risk prediction, but his pattern of alcohol consumption increases, characterizing alcohol abuse. Excessive alcohol use indicates that the Seek Mode is more activated through a memory of reward related to relaxation and calmness. Although the individual may achieve the state of low-risk prediction for a period of time, alcohol withdrawal leads to the activation of the Alarm Mode again. During withdrawal, the patient develops craving, which can be considered an internalizing (thoughts and emotions) symptom of the increased Seek Mode activation. Each point of the graphic represents the main symptoms of the disorder along the evolution. This cycle leads to alcohol dependence, which is also associated with other externalizing symptoms such as aggressive behavior. In this phase, the patient may get involved in situations of violence and develop psychiatric comorbidities as a consequence of substance use. The association of other drug use disorders, like opiate use, may lead the patient to an overdose.

Figures 5, 6 show examples of addiction progression and increasing reward prediction, respectively, from the point of view of the DREXI3 model. Figure 6, in particular, describes a possible trajectory of chronic substance use in which reward prediction increases with the use of more intense substances or stimuli in order to activate the Seek Mode despite future impairments, precipitating delay discounting and risky behaviors.

### 4.1 Appetite, sleep, energy, libido

The first dimension, abbreviated as appetite, sleep, energy, libido (ASEL), accounts for neurophysiological symptoms related to acute and chronic stress even in the absence of psychiatric symptoms. The relationship between disturbed sleep and addiction or psychiatric disorders is well supported by the literature. Sleep disturbances are one of the first symptoms related to stress and can be present in several mental illnesses as well, e.g., mood and anxiety disorders, psychosis, eating disorders, personality disorders. However, reduced sleep can also predispose to the development of psychiatric symptoms (78). The connection between sleep and mental illness has been analyzed in polysomnographic studies that reported sleep alterations in most disorders (79). Some studies have used models of allostasis to evaluate circadian rhythm, an essential element of physiological balance (80). Finally, addictive behaviors have been linked with adverse sleep outcomes. For instance, gaming disorder and problematic internet use have been associated with reduced sleep duration, subjective insomnia, and poor sleep quality (81–83).

Changes in appetite are linked to other physiological aspects, including sleep. The association between ghrelin (an orexigenic hormone), appetite, sleep, and depressive symptoms has been described. Some clinical findings support the hypothesis that ghrelin levels are increased in patients with hypersomnia, as both hypersomnia and hyperphagia are present in depressive episodes with atypical characteristics according to DSM-5-TR (77, 84). Even though appetite disturbances are common in different psychiatric syndromes, it is difficult to establish a causal relationship between them. Changes in diet can affect mood, while mental illness can impact eating habits. In addition, some common factors, such as genetic predisposition, stress, and other environmental cues, can lead to both appetite modifications and mental disorders (85). When it comes to appetite and addiction, there is increasing evidence supporting a common basis for obesity and some substance use disorders (86). Appetite-regulatory peptides, such as ghrelin and leptin, can play a role in addiction development and are expected to be useful in future treatment perspectives (87–89).

Energy alterations can also be present in different psychiatric disorders, including addictions. The most typical example is the use of stimulant substances leading to increased energy, euphoria and other symptoms through adrenergic activation (90). The same is true for libido, which can be affected by acute and chronic use of psychotropic drugs. Moreover, there is a discussion about how to classify compulsive sexual behavior, as it shares some features with other addictive behaviors (91). However, as these neurophysiological symptoms can be present in distinct disorders, their association with addiction may be more complex to analyze.

### 4.2 Impulsivity

Impulsivity is a core feature of addictive disorders, and therefore it comprises the second dimension in this proposed framework. It has been studied as a risk factor for the development and maintenance of these disorders (92, 93) and is a characteristic of the psychopathology of some addictive behaviors (94). The instrument most widely used to analyze this feature is the Barratt Impulsiveness Scale (BIS-11), which separately assesses attentional impulsiveness, motor impulsiveness, and non-planning impulsiveness (95). Non-planning impulsiveness, characterized by behaviors more oriented to the present than to the future, is particularly related to the concept of delay discounting. Initially derived from behavioral economics, this concept shows how some individuals discount delayed reinforcers by choosing immediate reinforcers, even though the latter are less worthy (96). Literature has described delay discounting as a marker of addictive behaviors. Therefore, this individual trait could help identify individuals at risk of developing an addiction and also influence the course of the disease and its severity, not to mention treatment choices (97, 98).

### 4.3 Compulsion

As discussed above, impulsivity can be considered an externalizing dimension associated or not with craving, which is an internalizing phenomenon. The same happens with compulsivity, which can also be triggered by craving – another core feature of addiction (99, 100). Compulsion, as the other dimensions of the model, is a transdiagnostic concept that can be observed in other syndromes, such as obsessive-compulsive disorder – researchers have studied brain structural changes present both in substance use disorders and obsessive-compulsive disorder (101). In the field of addiction, studies have started to focus on how compulsivity manifests across different types of addictive behaviors. A systematic meta-review has highlighted the importance of compulsivity-related neurocognition across alcohol use disorder and gambling. Nevertheless, there is a trend in those studies to understand compulsive behaviors not only as a consequence of chronic drug exposure, but as a relevant construct for addiction development (102). Another controversial topic in research is how precisely compulsivity can be defined, since it is expressed in distinct disorders. A recent systematic review highlights the need to refine compulsivity scales to achieve a more accurate analysis of these symptoms (103).

### 4.4 Anxiety and anger

Anxiety and anger symptoms have been included in a separate dimension. Typically, anxiety disorders have been associated with internalizing phenomena and externalizing responses, such as fight or flight (76), and personality traits, such as neuroticism. The association between social anxiety and substance use has been demonstrated. Social anxiety was positively related to alcohol problems in a meta-analysis that focused on college students (104). In alcohol use disorders, anxiety has been studied as part of a negative affect state during abstinence that increases the risk of relapse. A systematic review including animal and human studies identified a role played by the anterior insula in the abstinence network. In humans, the anterior insula is connected to the amygdala (known to be associated with anxiety and addiction) and to the nucleus accumbens (a known component of the abstinence network) (105). For instance, evidence supports a high prevalence of comorbid cannabis use and anxiety disorders, but more research is needed to establish a causal relationship between these conditions (106, 107).

In this framework, anger symptoms were placed in the same dimension as anxiety since they are usually correlated in clinical practice. However, anger and irritability can be present in other mental health conditions and personality disorders. A recent meta-analysis showed higher rates of anger scores among psychoactive substance users when compared to non-users (108). This finding highlights the relevance of anger in the evaluation and treatment of addiction.

### 4.5 Mood/depression

As predictions become worse, animals in general tend to present behavioral shutdown or hopelessness responses, and mammals in particular show signs of depression. Therefore, alterations related to mood, e.g., depressive and manic symptoms, were grouped in a different dimension in this proposed framework. The comorbidity of addiction and mood disorders has been extensively investigated. A review that analyzed the association between alcohol use disorder and major depression confirmed the high prevalence of comorbidity when one of the disorders is already present. Even though that study suggests a causal relationship in which alcohol use disorder increases the risk of depression, more research is needed to define the precise mechanism of the association (109). The interaction between depression and smoking has also been widely studied. Smokers with comorbid depression are less likely to quit when compared to non-depressive smokers. A systematic review that analyzed the mechanisms behind these lower cessation rates indicated low positive affect, high negative affect, and cognitive impairment as important underlying factors. These findings corroborate the need for treatment strategies focusing on negative internal states (110). Other psychoactive substances have been associated with depression. The use of cannabis during adolescence has been linked to a higher risk of developing depression in young adulthood (111).

### 4.6 Consciousness, attention, sensoperception, orientation, memory, intelligence and speech

The last dimension comprises changes in consciousness, attention, sensoperception, orientation, memory, intelligence, and speech, commonly associated with acute drug abuse and chronic or severe addiction. Both intoxication with and withdrawal from some substances can cause alterations in consciousness, orientation, and attention, e.g., delirium tremens (112). Psychotic symptoms, in turn, seem to be associated with severity of the substance use disorder and the pharmacological drug profile. Literature has focused on the acute use of cannabis, cocaine, and methamphetamines as a cause of psychotic episodes. Nevertheless, more studies are necessary to evaluate the role played by other substances, e.g., synthetic drugs, in this scenario (113). Acute cannabis use can also affect cognitive functions, including learning, memory, attention, and language; further studies should analyze the neuropsychological impact of chronic use. There is also concern about the risk of cannabis users developing psychotic episodes and even schizophrenia. Although multiple elements are known to be involved in the etiology of these disorders, cannabis use can be considered a risk factor for psychosis (114).

## 5 Discussion

The DREXI3 model described in this paper proposes an integrative, dynamic, transdiagnostic model for the development and maintenance of addiction. By focusing on the three homeostatic modes of the mind as a central aspect in the pathophysiology of these conditions, the authors aim to explain the process of addictive behaviors drawing on previous research into the role of emotional and behavioral regulation in these disorders (115). The present model proposes that a new paradigm of addiction, based on three modes of the mind, would allow patients to have a better understanding of the basic emotions and behaviors implicated in this complex condition, and could be useful for clinical evaluation and treatment planning.

The neurobiological basis of addiction, especially the reward system, has been widely investigated in recent years (37, 69, 99). However, evidence showing the influence of environmental factors and stress on addiction has led to the development of new frameworks (116). Some theories have focused on specific constructs, like attachment, to explain why some individuals are more prone to addiction (117). Other models argue that the emphasis on the disease model has diverted attention from other factors associated with drug use, and support that the development of addictive behaviors is a way of learning and responding to the environmental context, which may include traumas. The authors suggest that learning models may enable changes in therapeutic interventions, as the patient takes a more active role in the treatment (29).

It is clear that addiction comprises a complex group of disorders and results from multiple factors, including environmental contingencies that interact with individual neurobiological features. For instance, the role of stress in addiction has been well-documented: it can predispose to drug use via the action of stress hormones, such as the corticotropin-releasing hormone, which activates the amygdala and reduces activation of the hippocampus and prefrontal cortex, weakening executive control and increasing negative emotions (64). Environmental conditions, including stressful events, can influence the epigenetic response to drug use and predispose to the development of a disorder (70). It has been proposed that stress related to early life adversity can increase vulnerability to addictive behaviors by causing emotional and cognitive dysregulation (116). Moreover, a history of early life adversity, such as childhood neglect, may be related to symptom severity in addictive disorders (118). Overall, there is growing research correlating stress and emotional regulation with the neurobiological alterations that contribute to the process of addiction.

Addictive disorders are connected to the present framework because some of the main features of these disorders are the seeking behavior and the negative emotions that can be present in withdrawal and contribute to perpetuating the addiction cycle. According to the model, patients with substance use disorders present hyperactivation of the Seek Mode that leads them to present high levels of impulsivity and compulsivity in order to obtain reward. Impulsivity and compulsivity have been studied across different types of addiction. Some substances, like alcohol, are directly associated with brain damage and executive dysfunction, which worsen self-control toward future rewards (119). Nevertheless, studies have suggested that impulsivity is not only a consequence of chronic drug use, but may in fact, in many cases, underpin the development of substance and behavioral addiction (102). Conversely, an individual in Alarm Mode may present symptoms typically associated with depression and anxiety, which can be triggered by environmental factors in the present or the past, or even by drug withdrawal.

The evaluation and management of these symptoms, using various therapeutic interventions and approaches, could help prevent the occurrence of dysfunctional behaviors aimed at reestablishing the Balance Mode, probably reducing the chance of relapse, unless the mind predicts new risks or rewards related to the addiction behavior. For example, individuals who typically enter the Alarm Mode may benefit from interventions focused on symptoms like depression, anxiety, and irritability. Cognitive-behavioral interventions may be useful for the management of depressive and anxious symptoms (120, 121). Specific interventions, such as anger management and mindfulness-based cognitive-behavioral therapy, may be used to reduce anger and irritability (122), and there are specific interventions directed to the Seek Mode, targeting impulsivity and compulsive behaviors. Cue-exposure therapy can be considered an example of neuropsychological rehabilitation and has shown effect in reducing relapse (123). Mindfulness and other third-wave therapies have been seen as options to treat substance use disorders by acting on feelings related to craving (124, 125); contingency management may be a way of addressing the compulsivity component (37); mindfulness-based interventions may also be used in individuals in Balance Mode to enhance well-being and improve behavioral regulation (126). Overall, the treatment based on these three modes and their dynamic involves understanding individual functioning and values that shape predictions, and not only the symptoms of addiction.

As addiction represents a heterogeneous condition, a model based on different dimensions would enable therapists to perform a more personalized assessment. The dimensions proposed by the authors represent the main psychiatric syndromes already described in categorical systems (77) and include the core features of addictive behaviors (15, 102, 127). Notwithstanding, the authors acknowledge the need to develop adequate instruments to evaluate and quantify symptoms in each dimension. At present, one of the most widely used instruments to evaluate addiction is the Addiction Severity Index-6 (ASI-6), first developed in 1980 and now in its sixth version (128, 129). In addition to evaluating the severity of addictive disorders, new instruments should analyze their relationship with emotional and behavioral dysregulation.

Many theories developed to explain addiction have focused on the common features underlying different types of addictive disorders. Substance use disorders and behavioral addictions have similarities, but the manifestations of each illness differ (130). Considering these hypotheses of underlying factors contributing to different addictive behaviors, it has been proposed that the treatment of addiction should be transdiagnostic (37). In the present framework, the authors acknowledge that some interventions may be applied to a wide range of symptoms, while others may have to be customized according to individual needs. Furthermore, recent papers citing the self-medication hypothesis suggest that homeostatic theories may play a role in the addiction field (131–133). These theories tend to be more integrative, comprising different factors that may lead to the development of addiction and potentially contributing to the development of therapeutic interventions in the future. According to Feingold and Tzur Bitan, addiction psychotherapy should have an integrative approach, addressing both psychological and social aspects (134).

Disagreements about how to conceptualize substance abuse have been present in the literature for many years (135). Despite the continuous development of addiction theories and neurobiological research, some gaps remain when applying this knowledge to clinical practice. The perspective that new therapeutic strategies will derive from neuroscience discoveries draws from the concept of addiction as a brain disease. Notwithstanding, there is a trend of considering different theories when trying to understand addiction, so it seems unlikely that a single theory could explain the varying manifestations of this disorder. A more integrative framework may contribute towards a new understanding of addiction while raising new questions that are essential for the development of the field (33).

Considering addiction beyond its neurobiological basis may allow patients to take on new roles in therapeutic approaches. Individuals with an addiction may show biases in their choices, prioritizing maladaptive regulation rewards. However, they can still be sensitive to reward contingencies. Therefore, research into addictive disorders should consider therapeutic strategies involving more adaptive reinforcers, with patients playing a more active role in treatment (41). Furthermore, it is important to develop a model that considers the evolutive perspective of the systems that shape addictive emotions and maladaptive behaviors, in order to approach the phenomena in a dynamic way and translate findings to clinical practice and into effective public policies. Also, a model that includes evolutionary aspects could be more easily tested in animal research. The authors of this manuscript believe that the study of neurobiological aspects and systems related to the dynamic of the three modes as influenced by the environment could facilitate the validation of this addiction paradigm with animal models and its transposition to human externalizing and internalizing phenomena, in an attempt to minimize the translational crisis in the field.

McLean and Rose suggest that the compulsive, chronic, relapsing hallmarks of substance addiction can be explained through neurochemical maladaptations, and that these behaviors are in part the result of associations established with rewarding cues present in the environment. Such associations, combined with meanings given to experiences, could be represented in drug memories and memory systems, which those authors refer to as “neuro social relations.” They defend that a possible way of addressing the translational crisis would be by improving the understanding of these relations (136). The DREXI3 model accounts for these aspects by valuing the role of memory and its interaction with the environment to evaluate risk and reward. Furthermore, a transdiagnostic treatment approach to addictive disorders could have several benefits compared to the most common therapeutic strategies by focusing on the regulation of specific symptoms rather than on stopping drugs or other stimuli.

In conclusion, the conceptualization of addictive behaviors in association with cognitive and emotional responses that influence choices in a dynamic process involving modes and systems of the mind highlights the importance of cognitive function and decision-making over a narrow focus on the classical reward circuitry. The DREXI3 model described in this paper was developed using an integrative approach to the understanding of addiction, mainly based on a homeostatic purpose of the mind and aiming to be less stigmatizing and to improve the range of effective approaches based on the best scientific evidence to these behavioral disorders. According to this framework, addiction treatment should also be integrative and tailored to the needs of each individual. Previous research supports the influence of stress, emotional regulation, and environmental factors in addictive disorders. In the present framework, that knowledge is integrated with the hypothesis that the mind can work in three different modes, according to levels of stress and risk prediction.

Finally, the DREXI3 model proposed in this manuscript represents an innovative and potentially transformative framework for addiction understanding and treatment. By prioritizing cognitive function and decision-making processes and emphasizing the role of emotional, learning feedback and stress-related responses, this model offers a comprehensive lens through which addiction can be viewed. It is supposed to be a shift from the conventional focus on reward circuitry. The holistic approach it embodies looks for a reduction in stigma and an increase in effective, personalized treatment approaches. For the scientific community, this model could serve as a platform for future research, especially in exploring new treatment strategies that are tailored to individual needs. The understanding of addiction as a heterogeneous condition that can present with varying symptoms may foster the development of new treatment strategies in future research. Clinicians stand to benefit from the model’s integrative approach, potentially enhancing the therapeutic outcomes for patients. Moreover, patients themselves could gain a deeper insight into the nature of their condition, leading to more informed choices and better management of their own treatment paths. However, authors will encourage continued debate about possible limitations of this theoretical framework which needs to be explored and refined in order to substantiate its further applications. Considering the constant evolution of science, these possible limitations may be revised and integrated in the future. Additionally, a deeper understanding of addictive disorders could reduce stigma and contribute to more effective public policies concerning drugs.

## Data availability statement

The original contributions presented in the study are included in the article/supplementary material, further inquiries can be directed to the corresponding author.

## Author contributions

BL: Conceptualization, Writing – original draft, Writing – review & editing. AS: Conceptualization, Writing – original draft, Writing – review & editing. MeC: Writing – review & editing. FO: Writing – review & editing. EGu: Writing – original draft, Writing – review & editing. TR: Writing – original draft, Writing – review & editing. JS: Writing – review & editing. MaC: Writing – review & editing. FP: Writing – review & editing. EGr: Writing – review & editing. RG-O: Writing – original draft, Writing – review & editing. LD: Writing – review & editing. FK: Conceptualization, Writing – original draft, Writing – review & editing.
